# Polymers and Biomaterials for Posterior Lamella of the Eyelid and the Lacrimal System

**DOI:** 10.3390/polym16030352

**Published:** 2024-01-28

**Authors:** Kevin Y. Wu, Jamie K. Fujioka, Emilie Goodyear, Simon D. Tran

**Affiliations:** 1 Department of Surgery, Division of Ophthalmology, University of Sherbrooke, Sherbrook, QC J1G 2E8, Canada; yang.wu@usherbrooke.ca; 2 Faculty of Medicine, Queen’s University, Kingston, ON K7L 3N6, Canada; 3 Department of Ophthalmology, Faculty of Medicine, Université de Montréal, Montreal, QC H3C 3J7, Canada; 4 Centre Hospitalier de l’Université de Montréal (CHUM), Montreal, QC H2X 0A9, Canada; 5 Centre Hospitalier Universitaire Sainte-Justine, Montreal, QC H3T 1C5, Canada; 6 Faculty of Dental Medicine and Oral Health Sciences, McGill University, Montreal, QC H3A 1G1, Canada

**Keywords:** biomedical applications, biocompatibility, films and coatings, biopolymers, synthetic polymers, biomaterials, ophthalmology, oculoplastic surgery, lacrimal system, eyelid reconstruction, tissue engineering and 3D bioprinting

## Abstract

The application of biopolymers in the reconstruction of the posterior lamella of the eyelid and the lacrimal system marks a significant fusion of biomaterial science with clinical advancements. This review assimilates research spanning 2015 to 2023 to provide a detailed examination of the role of biopolymers in reconstructing the posterior lamella of the eyelid and the lacrimal system. It covers the anatomy and pathophysiology of eyelid structures, the challenges of reconstruction, and the nuances of surgical intervention. This article progresses to evaluate the current gold standards, alternative options, and the desirable properties of biopolymers used in these intricate procedures. It underscores the advancements in the field, from decellularized grafts and acellular matrices to innovative natural and synthetic polymers, and explores their applications in lacrimal gland tissue engineering, including the promise of 3D bioprinting technologies. This review highlights the importance of multidisciplinary collaboration between material scientists and clinicians in enhancing surgical outcomes and patient quality of life, emphasizing that such cooperation is pivotal for translating benchtop research into bedside applications. This collaborative effort is vital for restoring aesthetics and functionality for patients afflicted with disfiguring eyelid diseases, ultimately aiming to bridge the gap between innovative materials and their clinical translation.

## 1. Introduction

The application of biopolymers in the reconstruction of the posterior lamella of the eyelid and the lacrimal system marks a significant fusion of biomaterial science with clinical advancements. In this review, we aggregate research spanning from 2015 to 2023, offering a synthesis of knowledge that intersects innovative material applications with the nuanced requirements of oculoplastic surgery. This review provides a thorough understanding of the anatomical and functional exigencies of the eyelid and the lacrimal system, and critically evaluates the myriad of biomaterials and biopolymers currently employed and under investigation. 

We examine the intricate processes involved in repairing the delicate structures of the eyelid following trauma or disease, specifically focusing on the challenges associated with reconstructing the posterior lamella. Various surgical approaches to eyelid reconstruction are reviewed, taking into account the functional and aesthetic outcomes of anterior and posterior lamellar repairs. This review navigates through the indications for surgical reconstruction, the pivotal role of the posterior lamella in ocular physiology, and the guiding principles for choosing appropriate reconstructive techniques.

A critical analysis of the current standards in biomaterial applications is presented, juxtaposed with alternative interventions in posterior lamellar reconstruction. We scrutinize the properties of ideal tarsal substitutes, advocating for the harmonization of mechanical strength and biocompatibility to minimize the risks of inflammatory responses and to ensure integration with native tissues. Emerging biomaterials in this domain, such as decellularized grafts, acellular dermal matrices, and natural polymers, are explored in detail. Their applications, advantages, and limitations are systematically reviewed to delineate their potential and effectiveness in clinical practice.

Furthermore, this article delves into the lacrimal system’s anatomy and physiology, elucidating the function of the lacrimal drainage system and the implications of its obstructions. We discuss the indications for the use of nasolacrimal stents, their material properties, and the challenges of achieving both short-term and long-term success with these interventions. The potential of biopolymers to facilitate lacrimal gland tissue engineering is also a significant aspect of this review. Innovations in scaffold and hydrogel technology offer promising avenues for the regeneration of these tissues, with the goal of addressing dry eye diseases resulting from lacrimal gland dysfunction. The application of advanced manufacturing techniques, such as 3D bioprinting, is recognized for its transformative potential in creating organoids that emulate lacrimal gland function, opening new possibilities for both research and clinical applications.

The novelty of this review lies in its unique clinical insights and the establishment of a link between material science and clinical practice. It will concisely detail the structure-property relationships of biopolymers and synthetic polymers, targeting their application in posterior lamella eyelid reconstruction and lacrimal drainage system procedure. Prior literature has touched upon various aspects of biopolymer applications in ocular reconstruction [1]; however, a gap often exists in the translation of these materials from bench to bedside. Our review bridges this divide by integrating clinical perspectives, enabling a comprehensive view that spans foundational science to practical application. We elucidate the barriers to clinical translation, engage in a discourse on the current shortcomings, and posit directions for future research. 

## 2. Use of Functional Biomaterials in the Reconstruction of Posterior Lamellar Eyelids and Progress in the Creation of Tarsal Substitute

### 2.1. Anatomy of Marginal Eyelid 

**Figure 1:**
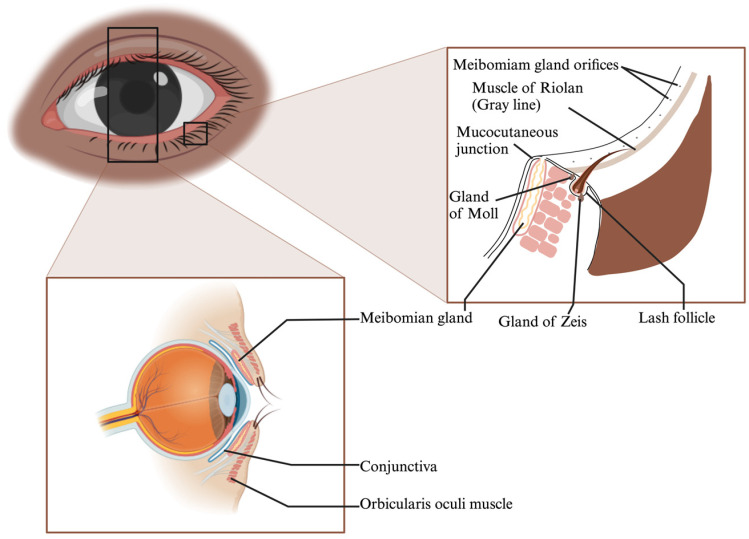
Eyelid Margin Structures: Meibomian Glands, Muscle of Riolan, Mucocutaneous Junction, Glands of Zeis and Moll.

The eyelid, a crucial anatomical structure, can be divided into the anterior and posterior lamella. The anterior lamella is made up of the skin and orbicularis oculi muscle, which supplies blood to the lamellar structures (Figure 1). The skin is thin, highly elastic, and void of subcutaneous fat, enabling smooth eyelid movements. The orbicularis oculi muscle, with its various subunits, enables eyelid closure. Defects in the anterior lamella can be repaired using adjacent tissues.

The posterior lamella, on the other hand, includes the palpebral conjunctiva and tarsal plate, which are critical for structural support and corneal protection [2,3,4,5]. The tarsal plate is a unique semilunar-shaped connective tissue that mirrors characteristics of cartilage and dense fibrous tissue [3,5] (Figure 1). Its extracellular matrix (ECM), rich in collagens and glycosaminoglycans, gives the eyelid its mechanical strength [6,7,8].

Embedded in the tarsal plate are the meibomian glands, which secrete meibum, vital for tear film composition [9,10]. Malfunctions in these glands can lead to ocular discomfort and diseases like evaporative dry eye [10].

**Figure 2:**
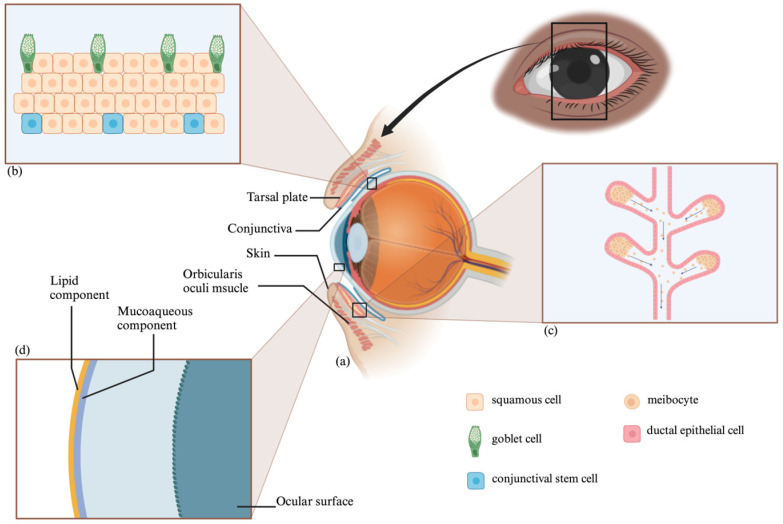
Eye Anatomy and Histology: Periocular Structures (**a**), Conjunctiva (**b**), Meibomian Gland (**c**), and Tear Film Layers (**d**).

The conjunctiva overlays the inner eyelid surface and comprises a stratified epithelium and vascularized basement membrane [11,12]. It houses goblet cells that secrete soluble mucins, crucial for maintaining the tear film [12,13] (Figure 2). Conjunctival loss can cause eyelid misalignment and dry eye symptoms [13] but can spontaneously re-epithelialize due to the presence of conjunctival stem cells [14].

In summary, the posterior lamella plays a dual role of providing mechanical support and ensuring sufficient corneal lubrication. The tarsal plate helps maintain the eyelid’s form and prevents abnormalities, while the conjunctiva and its embedded goblet cells, along with the meibomian glands, ensure tear film stability. An ideal posterior lamellar substitute should therefore possess mechanical strength, support efficient epithelial repopulation, and retain secretory functions.

### 2.2. Overview of Marginal Eyelids Defects and Reconstruction

Eyelid defects may result from tumor resection, trauma, or congenital disorders, requiring complex reconstruction due to the eyelids’ intricate anatomy and vital functionality. The main priorities in eyelid reconstruction include maintaining eyelid function, creating a stable eyelid margin, ensuring adequate eyelid closure for ocular protection, and optimizing aesthetic outcomes [15].

The selection of the reconstruction technique is contingent on several factors, such as the patient’s age, comorbidities, the size and location of the defect, and the surgeon’s preferences [16]. The guiding principles of eyelid reconstruction include reconstructing either the anterior or the posterior lamella with a graft, with the other providing a blood supply, and matching similar tissues.

Defects not involving the eyelid margins, whether small to moderate, can often be repaired via direct closure depending on the defect size and the tension of closure. Larger defects may require more intricate procedures like local skin flaps, grafts or transposition [16].

For eyelid margin defects, the reconstructive approach varies depending on the defect’s extent. Small defects often lend themselves to primary closure, while larger ones may require more complex strategies such as tissue advancement and flaps, free tissue autograft, or eyelid-sharing techniques [17,18,19]. In the context of the anterior lamellar region of the eyelid, these surgical techniques have proven successful if adherent to the ‘like for like’ principle, which advocates for a close match between defect and donor tissue characteristics [16]. Conversely, reconstruction in the posterior lamellar region remains a challenge [20,21,22]. The distinct anatomical, structural, and functional aspects of the eyelid, combined with the scarcity of suitable donor tissues, pose significant hurdles for conventional surgical techniques.

### 2.3. Indications for Reconstruction of Eyelids

The reconstruction of eyelids requires careful consideration of several factors, including the degree of defect involvement. Typically, defects smaller than 33% of the total width of the eyelid (i.e., minor defects) do not require reconstruction and can usually be treated with primary closure with or without lateral canthotomy or superior cantholysis. However, when defects exceed 33% of the total eyelid width, primary closure will inadvertently create excessive tension. This situation necessitates reconstructive procedures, including various types of grafts and flaps.

### 2.4. Current State—Current Gold Standard, Alternative Options, and Types of Biomaterials in Posterior Lamellar Eyelids Reconstruction

#### 2.4.1. Autogenous Tissue Utilization: Grafts and Flaps from the Eyelid and Periocular Region

Moderate lower eyelid defects (between 33% and 50%) necessitate semicircular flaps, or adjacent tarsoconjunctival flap with full-thickness skin graft. Larger defects (more than 50%) typically require eyelid-sharing techniques [23,24,25]. Those include procedures like the Cutler–Beard flap and the modified Hughes flap, which necessitate a temporary blockage of the visual axis, requiring a second surgery to reopen the eyelid [26,27,28,29]. Particularly in pediatric patients, this second procedure carries the additional risks inherent in general anesthesia, which is a crucial consideration in children. Furthermore, these procedures often lead to an upper eyelid that is thick and exhibits reduced mobility. It is also worth noting that such temporary occlusion of vision is particularly suboptimal for patients with only one functional eye and for children, where there is a risk of inducing deprivation amblyopia. Other types of flaps, such as the median forehead flap and the Mustardé flap, can also be utilized for larger defects. While these methods offer benefits of preventing temporary occlusion of the eye, they still require only two surgical stages [30,31]. 

A free tarsoconjunctival graft taken from the contralateral eyelid covered with a skin–muscle flap avoids the need of second surgery. However, its drawbacks include limited graft size, donor eyelid morbidity, the potential for eyelid retraction, and scar formation. These complications can lead to conditions such as ectropion and entropion [32,33]. This technique also necessitates the presence of adequate redundant upper eyelid skin. This skin forms a local myocutaneous flap to cover and supply blood to the graft. Therefore, individuals without sufficient redundant skin may not be ideal candidates for this procedure [34,35]. Additionally, preserving the native structures of the lid margin (i.e., eye lashes and functional meibomian glands) presents another drawback. As a potential solution, the tarsomarginal graft, which is a composite graft composed of the tarsal plate, conjunctiva, lid margin, and eyelashes, has been proposed. However, the high complication rates associated with the tarsomarginal graft significantly hinder its clinical application [36]. 

#### 2.4.2. Autogenous Tissue Utilization: Grafts from Other Regions

**Figure 3:**
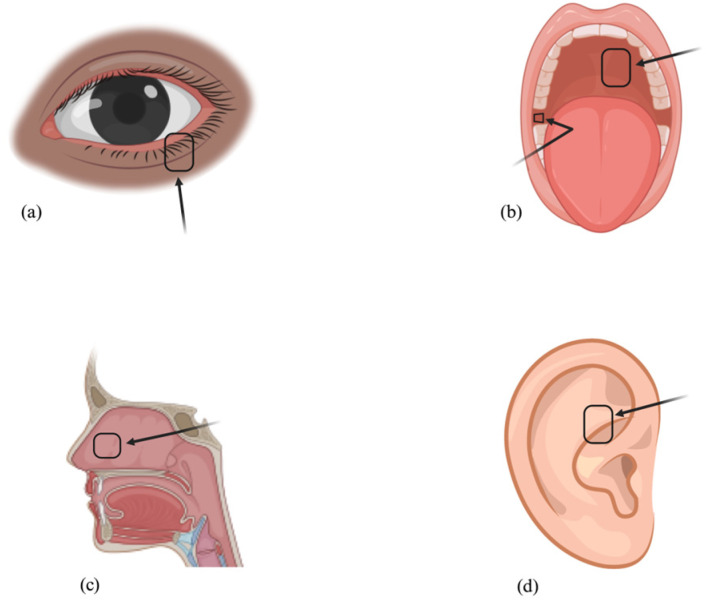
Mucous Membrane Autograft Donor Sites: Lower Eyelid (**a**), Buccal Mucosa (**b**), Hard Palate (**b**), Nasal Septum (**c**), and Auricular Cartilage (**d**).

To circumvent the limitations of autografts derived from the posterior lamella, clinicians have explored autografts sourced from other regions, such as the lip [37,38], buccal mucosa [39,40], gingival alveolar mucosa [41], hard palate [42,43], auricular cartilage, and nasal septum [44,45] (Figure 3).

Oral mucosa grafts, comprised of vascular connective tissue and avascular epithelium, are known for their resilience and high vascularization, favoring graft integration post-transplantation [46]. They serve as effective conjunctival replacements [39,47]. The absence of goblet cells, however, can lead to corneal irritation and dry eye symptoms [48]. They also lack the physical rigidity that a native tarsal plate can provide [47].

Hard palate mucoperiosteal grafts, bearing a structural similarity to the posterior lamella, provide both structural support and a moist mucosal surface [43,49,50]. Yet, post-transplantation persistence of orthokeratosis and/or parakeratosis can cause corneal irritation and discomfort [51]. Moreover, the donor site must be left to heal secondarily (it cannot be primarily closed), making this a much more painful procedure with an extended post-op healing period [51].

Nasal mucosal grafts share a histological resemblance to the tarsoconjunctiva, containing a large number of goblet cells and subepithelial mucin glands [51], facilitating mucin substitution. These grafts provide firmness and a stable eyelid margin [52,53]. However, grafts can contain nasal hair causing conjunctival or corneal irritation [54].

Auricular cartilage grafts offer appropriate flexibility and strength [55,56]. Due to a low metabolic rate and minimal vascular requirements, they remain viable for many years post-implantation [55,56]. Yet, their rough surface may cause corneal irritation. They also lack secretory function [57].

Despite the range of nontarsoconjunctival autograft options available for posterior lamellar reconstruction, there remain challenges including incomplete functional matching, limited donor area availability, and donor-site morbidity. These have prompted the exploration of alloplastic biomaterials and substitute for posterior lamellar reconstruction [58].

### 2.5. Ideal Properties of a Tarsal Substitute

A tarsal substitute for posterior lamellar tarsal plate should possess several ideal attributes:

- Structure: Should be thin and stable.
- Biocompatibility: Should be highly biocompatible.
- Integration: Should have the ability to seamlessly merge into the peripheral tarsus.
- Inflammatory Responses: Should not provoke any inflammatory responses.
- Mimicry: Should mimic the physical structures and biological functions of the native extracellular matrix.
- Cell Support: Should foster cell survival, proliferation, and growth.

Today, the major biomaterials leveraged for posterior lamellar reconstruction encompass decellularized extracellular matrix, natural polymers, and synthetic polymers..

### 2.6. Emerging Biomaterials and Their New Applications in Posterior Lamellar Eyelid Reconstruction

An overview of the key features, advantages and disadvantages of emerging biomaterials used in posterior lamellar eyelid reconstruction is described below and summarized in Table 1. 

#### 2.6.1. Amniotic Membranes

The innermost layer of the placental sac, known as the amnion, is a naturally acellular biomaterial that can be collected for the purpose of aiding injured tissue, safeguarding and fortifying defects against further deterioration, and facilitating the process of recellularization. Recent research has demonstrated that the composition of amniotic membranes can induce conjunctival epithelization by acting as a scaffold for epithelial cell growth with anti-angiogenic, anti-inflammatory, and anti-scarring properties [58,59]. Variations, including freeze-dried, dehydrated and urea-de-epithelialized amniotic membranes, have been optimized for this purpose [60,61,62,63]. In 2017, Agraval and colleagues conducted a retrospective review of 53 patients who underwent excision of conjunctival cancerous lesions with fresh frozen amniotic membrane grafts. Among the cohort, 22.7% had local surgical complications including scarring, symblepharon, granuloma, and eye movement restriction. No metastases were found at the graft site, leading the authors to conclude that amniotic membrane grafts improved surgical conjunctival outcomes by enhancing healing, diminishing scarring, and enabling broader surgical margins [64]. 

Similarly, Palamer and team analyzed 10 patients with conjunctival melanoma who underwent total excision, cryotherapy to surgical margins, and ocular surface grafting using cryopreserved amniotic membrane. Their study observed mild long-term complications such as limbal stem cell deficiency (2 eyes) and subclinical symblepharon (3 eyes). Yet, this approach proved safe and effective in managing large conjunctival melanomas while allowing for wider tumor margin excision [63]. 

In another study, Miyakoshi and colleagues compared the histological dynamics and long-term safety of hyper-dry amniotic membrane (HDAM) to the Ambio2TM amniotic membrane graft when used for ocular surface reconstruction in rabbits. Both were completely absorbed without scarring within 6 months, though HDAM showed a higher rate of inflammatory cells 30 days post-surgery [61]. Overall, these membranes, when applied directly or in conjunction with other techniques, can support the growth of conjunctiva-derived cells and have demonstrated absorption without scarring [61,64,65,66]. However, their limited availability and potential risks, such as infectious disease transmission, challenge their widespread adoption [61].

#### 2.6.2. Decellularized Grafts

Decellularization is a targeted procedure that removes cells from organs or tissues, resulting in a cell-free scaffold made up of the tissue’s intrinsic extracellular matrix. This cell removal significantly reduces the chances of graft rejection by depleting the scaffolds of important histocompatibility complexes. Notably, decellularized grafts sourced from both porcine and human origins are gaining increasing attention in the field of ocular research. Adipose-derived mesenchymal stromal cells (ADMSCs) are a relatively abundant source of graft material rich in growth factors that can be acquired through minimally invasive means. Yan’s team used ADMSCs to craft a matrix (ADMA) to heal conjunctival defects in rabbit models. In their study, the fabricated ADMA demonstrated in vivo transplant viability, providing structural support without cosmetic deformities. Their matrix also proved superior to amniotic membranes in promoting faster wound closure and maintaining undifferentiated conjunctiva epithelial stem cells, critical for long-term conjunctival reconstruction. Additionally, ADMA enhanced epithelial stem cell proliferation by activating the Akt signaling pathway, making it a promising candidate for conjunctival substitutes in ocular reconstruction [67]. 

Zhao and colleagues introduced a decellularized porcine conjunctiva (DPC), eliminating major xenoantigens while preserving vital matrix components, in rabbit models. Similar to Yan’s findings, their approach showed superior transplant stability and epithelial regeneration compared to amniotic membrane [68]. In a clinical study, Shan and his team compared the outcomes of using autologous conjunctiva (AC), autologous oral mucosa (AOM) or DPC in various combinations to treat severe symblepharon in 16 patients. Their investigation showed that DPC used alongside AC or AOM resulted in complete treatment success in 75% of cases, partial success in 18.75% and failure in 6.25%. Thus, they concluded that DPC is a viable option to treat severe symblepharon, resulting in significant recovery in eye mobility and fornix depth especially when combined with autologous mucosa [69]. Chen’s group created enhanced decellularized porcine pericardium scaffolds for conjunctival reconstruction by crosslinking them with aspartic acid and human endothelial growth factor. Their biomaterial resulted in a closed, multilayer epithelium with goblet cells and minimal scarring in animal models [70]. In another innovative approach, Witt and his team combined human conjunctival explant with porcine decellularized conjunctiva to create a stable substitute rich in goblet cells, ideal for patients with significant conjunctival cell depletion [71]. 

TarSys^®^ is a clinically approved acellular membrane used for tarsal plate reconstruction, derived from decellularized porcine small intestinal submucosa, and contains Col I, Col III, Col IV, and related glycosaminoglycans. Liao and colleagues assessed the effectiveness of this biomaterial as an eyelid spacer graft to correct lower lid retraction in 32 Graves ophthalmology patients. Their results showed significant improvements in lower eyelid height and reduction in lagophthalmos, with no infections, corneal erosions, or need for further surgeries observed, indicating that decellularized porcine-derived membrane can be a valuable option for eyelid reconstruction [72]. However, being a xenogeneic product, there are concerns about potential infectious disease transmission and noted immunogenic inflammatory-related issues [72,73,74]. The combined efforts of these researchers exemplify the continuous advancement in conjunctiva and tarsal plate regenerative therapies, fostering for hope for improved treatments in this field. 

#### 2.6.3. Acellular Dermal Matrices (ADM)

Recent advances in periocular surgical procedures involve the use of various dermal collagen biomaterials in eyelid surgery. Specifically, acellular dermal matrices (ADM) sourced from human, bovine and porcine dermis offer ready-to-use biomaterials for tarsal plate replacement [75,76,77,78,79,80,81]. Structurally, ADMs are durable pliable sheets made of cross-linked collagen with its constituent elastin fibers that resist breakdown and reabsorption. Importantly, this biomaterial is structurally similar to human tissue, serving as a natural scaffold for epithelial migration, vascularization and fibroblast infiltration. They have been extensively used for abdominal mesh and hernia repair, breast reconstruction and burn treatment due to their low immunogenicity and high histocompatibility. Due to their pliability ADMs can adapt to different ocular shapes.

Clinical studies have shown the effectiveness and safety of these matrices as lower eyelid spacer grafts, which can augment the height of retracted eyelids similar to autologous grafts [79,81]. In situations where tarsal plate defects are paired with conjunctival deficiencies, ADM grafts can assist in reconstruction, allowing full conjunctival epithelialization within 3–6 weeks post-operation [82]. In a study of 12 patients (16 eyelids) using Permacol™, a decellularized porcine dermal membrane for lower eyelid retraction, McGrath and colleagues observed a notable reduction in the inferior scleral show and an average eyelid elevation of 0.91 mm over 8 months [78]. However, in a comparative study of Permacol versus buccal mucosal graft reconstruction in anophthalmic patients, the Permacol graft took longer to vascularize and experienced more shrinkage over time compared to hard palate mucosal grafts [83]. 

#### 2.6.4. Natural Polymers

Natural polymers are highly sought-after biomaterials in tissue engineering because of their exceptional biocompatibility, appropriate mechanical properties, and porous nature. They are advantageous because they do not release cytotoxic degradation products, are often processed using sustainable methods and their degradation rates can be easily manipulated by modifying formulations or processing conditions [84]. They are typically derived from ECM components, such as collagen (Col I), chitosan, and keratin, which serve as another biocompatible structural framework for conjunctival or tarsal defects [85,86,87,88]. In recent decades, a range of techniques for creating scaffolds from natural polymers has been developed, encompassing methods such as electrospinning, freeze-drying, and 3D printing.

Col I is the most abundant protein in the conjunctival matrix, and thus, is commonly used for conjunctival repair. In rabbit models, collagen-based materials have been shown to promote conjunctival regeneration with rapid re-epithelization, goblet cell repopulation, minimal fibrosis, and wound contracture [86,89]. Nonetheless, collagen’s mechanical characteristics frequently exhibit instability, leading research in the development of collagen-based scaffolds to emphasize enhancing both strength and bioavailability. To improve collagen hydrogel stability, Drechsler and team used compressed collagen to support human conjunctival cell expansion for fornix reconstruction, yielding promising results akin to amniotic membrane [88]. 

Chitosan, a linear polysaccharide, is renowned for its advantageous properties such as biocompatibility, bioactivity, and biodegradability. Diverse types of chitosan-based scaffolds exist, such as films, particles, hydrogels, fibers and sponges. Importantly, chitosan possesses unbound amino groups that can undergo protonation, rendering it adaptable for the incorporation of biochemical groups. This protonation capability facilitates electrostatic interactions with DNA, proteins, lipids, or negatively charged synthetic polymers. Sun and collaborators created chitosan hydrogel scaffolds mimicking eyelid tarsus tissue and assessed biocompatibility by applying human eyelid skin fibroblasts. Their results revealed that these scaffolds effectively supported the growth and proliferation of both mouse and human fibroblasts [85]. 

Combining both chitosan and collagen, Xu and team developed biphasic scaffolds by compositing both biomaterials into sponges of varying thicknesses onto a polymer network, simulating the natural anatomy of the posterior lamella. These scaffolds showcased approximately 90% porosity, appropriate degradation rates, and good biocompatibility, influencing cellular behaviors like proliferation and distribution. When tested in a rabbit model, the scaffolds encouraged re-epithelization, inferring its potential as a substitute for tarso-conjunctival repair [90]. 

Although natural biopolymers have made notable progress, challenges like insufficient vascularization, unpredictable degradation rates, and limited re-establishment of nerve connections in large grafts continue to impede the clinical adoption of these biomaterials. Consequently, there is an ongoing requirement to innovate new methods for creating pre-vascularized and re-innervated tissues, facilitating processes such as angiogenesis, neovascularization, and nerve integration to achieve the development of fully functional tissues [84]. Considering that it is improbable for a single polymer to meet all the criteria for constructing a scaffold to repair posterior lamella eyelid defects, the use of a combination of biomaterials is more likely to offer an optimal solution that aligns with both the cellular and mechanical requirements of these tissues. The ideal approach is to leverage the strengths of each material type and blend them together, representing a focal point for future research.

#### 2.6.5. Synthetic Polymers for Conjunctival Reconstruction

In contrast to natural polymers, synthetic biomaterials offer the advantage of processability that can be tailored to suit the requirements of specific tissues. For conjunctival repair, polymers such as poly(acrylic acid) (PAA) [91], poly(ε-caprolactone) (PCL) [91,92,93], poly(vinyl alcohol) (PVA) and poly(lactic acid) (PLA) [91] have been explored. Of these, PCL has not only received FDA approval, but also stands out for its promising potential in clinical applications. Bosworth and team used PCL and decellularized matrices in electrospun scaffolds for conjunctival defect repair, revealing improved cell layering [92]. Yao and team developed an ultrathin collagen and poly(L-lactic acid-co-ε-caprolactone) (PLCL) scaffold that, when tested in vitro with conjunctival epithelial cells, demonstrated strong cell growth and health, with notable porosity and suitability for treating conjunctival epithelial coloboma [94]. PLA is prominent in biomedicine but is inherently hydrophobic, limiting cell adhesion. However, Yan and team crafted a PLA-based scaffold enhanced with cellulose, silk peptide, and levofloxacin, which demonstrated effective conjunctival repair in rabbit models [95]. 

Synthetic substrates have drawbacks stemming from their structural differences when compared to in vivo cellular microenvironments. These disparities are due to mismatched orientation, distribution, and density of the necessary signaling cues required to promote tissue repair. Comparing natural biopolymers with synthetic electrospun scaffolds (PCL, PVA, and PAA), He and colleagues determined that natural biopolymers offered significant advantages over synthetic materials due to reduced immune response risk and better tissue integration. Despite some mechanical inconsistencies, natural biopolymers allowed for effective attachment and goblet cell growth. Conversely, synthetic electrospun scaffolds offered customization, but struggled with consistent and viable goblet cell growth. He and team concluded that biopolymers, particularly collagen hydrogels and modified silk films, outperformed synthetic polymers for conjunctival transplantation [91]. 

#### 2.6.6. Synthetic Polymers for Tarsal Plate Reconstruction

Recent studies have also explored the use of synthetic polymers, including porous polyethylene (PE) [90,96], poly(3-hydroxybutyrate-co-3-hydroxyhexanoate) (PHBHHx) [97], poly(lactic-co-glycolic) acid (PLGA) [98,99,100], and PCL [101] for tarsal plate reconstruction. Among these, only high-density porous PE (Medpor^®^) has been clinically leveraged as an eyelid spacer to address lower eyelid retraction. However, its application in eyelid surgery is limited by notable postoperative complications like instability, implant visibility, irregular skin shaping, and discomfort [102]. While alternative synthetic scaffolds for tarsal reconstruction have been researched in animal models, their clinical acceptance is hindered by poor tissue compatibility and implant-related inflammation. For example, Zhou and colleagues studied the application of a PHBHHx scaffold as a tarsal substitute in rat eyelid defects, revealing a blend of acute and chronic inflammatory responses with prominent inflammatory cell infiltration [97]. Alternatively, Gao and colleagues enhanced poly(propylene fumarate) (PPF) to match the tarsus’s mechanical needs and created porous scaffolds by copolymerizing it with the hydrophilic monomer 2-hydroxyethyl methacrylate (HEMA). Their research demonstrated that this PPF-HEMA complex exhibited satisfactory repair capabilities and biocompatibility, whole provoking minimal tissue responses in rabbit models [103]. 

#### 2.6.7. Cellular Approaches

Acellular methods commonly result in fibrotic tissue formation, primarily restoring mechanical support without addressing essential secretory functions. Consequently, recent progress is centered on developing cellular grafts that aim to fully reinstate both the structural integrity and functional aspects of the posterior lamellar. This innovative approach involves seeding bioscaffolds with cells that replicate desired tissue functions, subsequently facilitating transplantation to foster tissue regeneration [71,99,101]. 

A variety of cell types have been explored to foster this approach. For instance, xenogeneic conjunctival epithelial cells have been used to engineer conjunctival substitutes, preserving essential traits like mucin expression. However, xenobiotic components require extended laboratory cultivation, limiting their widespread clinical utility. Biomaterials, such as decellularized matrices and synthetic polymers, act as scaffolds for epithelial cells during lab cultivation, yet challenges persist regarding their surgical manageability and in vivo strength [12,71]. Since the nasal mucosa naturally contains goblet cells and amniotic epithelial cells capable of differentiating into both conjunctival epithelial and goblet cells under specific conditions, they have also been used to repair conjunctival defects [104]. In cases of significant conjunctival loss or scarring, oral mucosal epithelial cells offer an alternative. They have been used clinically in techniques like cultivated oral mucosal epithelium transplantation for repairing the ocular surface and forniceal structure. While these methods hold promise, particularly in forniceal repairs, the lack of goblet cells in the engineered oral epithelium limits its full potential [105,106].

In tarsal reconstruction, Chen et al. developed a biomimetic tarsal substitute by overlaying a 3D-printed PCL scaffold with a decellularized matrix from adipose-derived stem cells. In vitro tests demonstrated cytocompatibility and support for sebocyte growth. One month post-implantation in mice, the scaffold showed promising glandular structures and lipid secretion [101]. However, further research is required to validate the functional restoration of regenerated meibomian glands.

Zhong and colleagues pioneered an efficient in vitro expansion technique for rabbit-derived conjunctival stem cells and employed bioprinting to create injectable hydrogel constructs loaded with these cells, maintaining viability and differentiation capability. This innovative approach combined advanced cell culture and bioprinting, offering a potential solution for ocular surface disorders [107]. Similarly, Wu and collaborators created a functional scaffold using a decellularized matrix from rabbit subconjunctival fibroblasts and loaded it with conjunctival epithelial stem cells, which showed promise in rabbit models for ocular surface repair [14]. To achieve tarsal plate reconstruction, Dai and colleagues created a composite structure using a poly(lactide-co-glycolide) (PLGA) sponge, bone marrow-derived stem cells (BMSCs), plasmid DNA for transforming growth factor-β1, and fibrin gel. When implanted into rabbit tarsal plate defects, there was evident structural and functional repair within 8 weeks, including ECM deposition and meibomian gland formation. Although the best source of these glands remains debated, this study’s groundbreaking work on restoring the tarsus’s lipid secretion opens up fresh possibilities for posterior lamellar tissue engineering [99].

**Table 1:** Emerging Biomaterials in Posterior Lamellar Eyelid Reconstruction.

Material Type	Key Features and Application	Advantages	Challenges	References
Amniotic membranes	-Provides a basement membrane and avascular stromal matrix that can induce conjunctival epithelization -Placed directly over the tarsus up to the eyelid margin or in combination with other techniques -Widely used as a substitute for conjunctival replacement	-Low immunogenicity -Antimicrobial, antiviral, antifibrotic and antioangiogenic properties -Support growth of surrounding conjunctiva-derived cells such as non-goblet epithelial cells and goblet cells -Can be applied directly or in combination with other techniques for posterior lamellar reconstruction -Gets absorbed within 6 months without scarring	-Limited availability -Inconsistent tissue properties -Risk of infectious disease transmission and inflammatory-related complications	[58,59,60,61,62,63,64,65,66]
Decellularized grafts	-Cell-free scaffold sourced from porcine and human origins -Adipose-derived mesenchymal stromal cells (ADMSCs) are used as a graft material -Various decellularized grafts have been developed for conjunctival and tarsal plate reconstruction	-Reduced graft rejection due to removal of histocompatability complexes -Promotes faster wound closure and maintains undifferentiated conjunctiva epithelial stem cells -Enhanced epithelial stem cell proliferation through Akt signalling activation -Successful treatment of severe symblepharon with decellularized porcine conjunctiva (DPC) combined with autologous conjunctiva or oral mucosa -Effective use of TarSys^®^ for eyelid reconstruction, resulting in improved lower eyelid height and reduced lagophthalmos in Graves patients	-Concerns of infectious disease transmission with xenogeneic products -Immunogenic and inflammatory-related issues	[67,68,69,70,71,72,73,74]
Acellular dermal matrices (ADMs)	-Sourced from human, bovine and porcine dermis -Durable, pliable sheets made of cross-linked collagen and elastin fibers, structurally similar to human tissue -Scaffold for epithelial migration, vascularization and fibroblast infiltration	-Pliable material that can be adapted to ocular shapes -Low immunogenicity and high histocompatability -Safe and effective as lower eyelid spacer grafts -Promote conjunctival epitheliazation	-May take longer to vascularize than other materials -Potential shrinkage of material over time	[75,76,77,78,79,80,81,82,83]
Natural polymers	-Commonly derived from extracellular matrix (ECM) components like collagen I, chitosan and keratin -Serve as a biocompatible framework for conjunctival or tarsal defect repair	-Favoured for their biocompatibility, suitable mechanical properties and porous structure -Do not release cytotoxic degradation products -Processed with sustainable methods -Degradation rates are easily modifiable -Collagen-based materials, particularly Col I, promote rapid conjunctival regeneration with re-epithelization, goblet cell repopulation, minimal fibrosis, and wound contracture -Chitosan is biocompatible, bioactive, and biodegradable and can be adapted to incorporate various biochemical groups -Combining chitosan and collagen in biphasic scaffolds shows promise in supporting tissue growth and repair	-Insufficient vascularization, unpredictable degradation rates, and limited nerve connections in large grafts -Innovation is needed to create pre-vascularized and re-innervated tissues to achieve fully functional tissue regeneration -Combining multiple biomaterials to address both cellular and mechanical requirements is a promising approach for future research	[84,85,86,87,88,89,90]
Synthetic polymers	-Poly(acrylic acid), poly(ε-caprolactone (PCL), poly(vinyl alcohol) and poly(lactic acid) for conjunctival defects -Porous polyethylene (PE), poly(3-hydroxybutyrate-co-3-hydroxyhexanoate) (PHBHHx), poly(lactic-co-glycolic) acid, and PCL for tarsal plate reconstruction	-Processability that can be tailored to suit the requirements of specific tissues -Some synthetic scaffolds demonstrated strong cell growth and biocompatibility	-May have mismatched orientation, distribution and density of signalling cues compared to native tissue to promote effective tissue repair -May have greater immune response risk and suboptimal tissue integration than natural biomaterials	[91,92,93,94,95,96,97,98,99,100,101,102,103]
Cellular approaches	-Involves seeding bioscaffolds with cells that replicate desired tissue functions and facilitate transplantation for tissue regeneration -Various cell types have been explored including xenogeneic conjunctival epithelial cells, nasal mucosa cells, amniotic epithelial cells, and oral mucosal epithelial cells -For tarsal reconstruction, biomimetic substitutes are being developed using 3D-printed scaffolds, decellularized matrices, adipose-derived stem cells, and bioprinting techniques	-Can restore both structural integrity and functional aspects of the posterior lamella -Injectable hydrogel constructs loaded with conjunctival stem cells and other combinations of stem cells, plasmid DNA, and fibrin gel have shown promise in repairing ocular surface and tarsal defects	-Xenogeneic cell components may require extended laboratory cultivation, limiting clinical utility -Challenges persist regarding surgical manageability and in vivo strength of biomaterials and scaffolds -Functional restoration of regenerated meibomian glands and debate over the best cell source for these glands remain areas of research -Further validation and research are needed to confirm the effectiveness of cellular graft approaches	[12,14,99,101,104,105,106,107]

### 2.7. Challenges, Barriers, Gaps in Knowledge, and Future Directions

The utilization of biomaterials for the reconstruction of the posterior lamellar eyelid presents a promising but complex avenue of research. Historically, techniques for posterior lamellar reconstruction have been reliant on replacement strategies involving implanting autografts or cell-free biomaterials in the defective area for mechanical support. Various alternatives, such as auricular cartilage, buccal mucosa, and nasal septum, have been explored [108,109]. While such biomaterials are cost-effective and easily sourced, complications such as graft contraction, graft exposure, shrinkage, and cyst formation are common. Another notable drawback is their inability to offer a smooth epithelialized surface or restore secretory functions [83,110,111]. 

The potential of tissue engineering for posterior lamella reconstruction holds promise for ocular regenerative medicine, aiming to surpass the limitations of autologous approaches. While recent studies indicate that implanted decellularized matrices can promote cell growth, Chen and colleagues observed fibrosis rather than full re-epithelization in rabbit models [70]. Modern tissue engineering aims to bridge the gap between the biomechanical properties of traditional grafts, artificial allografts, and the native structures of the eyelid. Natural polymer-based scaffolds have gained traction in soft tissue repair, but current products often face limitations such as inadequate mechanical properties, uncontrolled degradability, and unfavorable immune responses. Addressing these challenges involves advanced processing methods to maintain bioactivity, tailored material design, advanced fabrication techniques and the incorporation of adhesion molecules [112]. Further, replicating or replacing the native functions of the posterior lamella remains a challenge. For example, though a modified PPF-HEMA biopolymer has been found to mimic the tarsus’s mechanics, achieving a perfect structural match remains elusive [103]. Alternatives include engineering tarsal equivalents using lipid-secreting sebocytes, yet their self-renewal and secretion capabilities remain suboptimal [101]. 

Achieving an ideal balance between mechanically supporting the eyelid and restoring secretory functions for re-epithelialization proves difficult in conjunctival repair. Restoring secretory function is challenged by the inability to regenerate goblet cells and meibomian glands, crucial for ocular surface lubrication [113]. The recent discovery of a Krox20-expressing stem cell population offers hope for meibomian gland regeneration [114]. Similarly, regenerative approaches involving BMSCs or conjunctival stem cells capable of regenerating epithelial and glandular tissue have emerged [67,107]. Ex vivo studies show successful delivery of high cell counts to target regions in animal models, suggesting potential for in vivo applications [107,115]. However, these methods are still in their infancy and require further development before advancing to human trials. There is a specific need to better understand long-term in vivo material behavior and how the biomechanics of artificial grafts compare to native tissues.

Future directions in the field must address these gaps through rigorous, long-term studies and explore the integration of novel technologies like 3D bioprinting. Recent advances include bioprinted tracheal, nasal cartilage, and long bone constructs, highlighting the potential to produce tarsal substitutes. Chen and colleague’s work on a biomimetic tarsal plate substitute using a 3D-bioprinted scaffold is one example of this approach. 3D bioprinting has also been utilized in conjunctival engineering, with 3D-printed gelatin-based membranes showing promising results. These membranes have outperformed traditional amniotic membranes in goblet cell density, degradation predictability, and minimized scar and inflammation responses [101]. This advancement suggests that 3D bioprinting could be pivotal in designing posterior lamellar substitutes that match the precise mechanical and structural requirements of native tissues. 

## 3. Use of Biopolymers to Address Diseases Affecting the Lacrimal System

### 3.1. Anatomy and Physiology of Lacrymal Drainage System

The lacrimal duct system serves as a conduit for tears from the eye surface to the nasal cavity and is categorized into upper and lower divisions. Obstructions within this pathway frequently lead to epiphora, or excessive tearing, affecting both children and adults. 

#### 3.1.1. Upper System: Lacrimal Puncta to Canaliculi

Tears enter the upper system via the lacrimal punctum, located at the mucocutaneous border of each eyelid. These punctae are strategically positioned to face each other when the eyelids close [116].

From the punctum, tears flow through the vertical canaliculus, which widens into the ampulla before making a medial turn and continuing horizontally along the eyelid. In a majority of individuals, the upper and lower canaliculi merge to form a common canaliculus prior to entering the lacrimal sac. A smaller subset has canaliculi that connect directly to the sac [116]. The valve of Rosenmuller delineate the common canaliculus from the lacrimal sac, ensuring a unidirectional flow of tears [117].

#### 3.1.2. Lower System: Lacrimal Sac to Intraosseous Duct

The lacrimal sac is situated anterior to the orbital septum within the lacrimal fossa, bordered by the lacrimal and maxillary bones [118]. Tendinous insertions from the orbicularis muscle aid in sac movement and tear propulsion [119]. The sac is divided into a fundus and body based on the insertion point of the common canaliculus. The lacrimal duct then extends from the sac, taking specific angulations in the coronal and midsagittal planes. The duct passes through the maxillary bone and ends at the valve of Hasner within the nasal cavity’s inferior meatus.

This intricate architecture enables the lacrimal duct system to effectively manage tear drainage, thereby preserving the ocular surface’s integrity, function, and comfort.

### 3.2. Overview of Tear Duct Stents

Nasolacrimal stents serve as instrumental devices in maintaining the patency of the tear drainage system. Over the years, various materials, including silk, nylon, polyethylene, and polypropylene, have been employed [120]. Currently, silicone-based tubes with an open central lumen are the standard, generally utilized for short-term intubation periods, although long-term applications do exist [121].

Nasolacrimal stents can be broadly classified into bicanalicular and monocanalicular types, based on the specific anatomical segments they intubate. Bicanalicular stents, forming a closed circuit, pass through both the upper and lower canaliculi, and can be configured to intubate varying segments, from the common canaliculus to the entire nasolacrimal drainage system [122]. Monocanalicular stents, in contrast, do not form a closed circuit and only intubate either the upper or lower canaliculus [123].

In certain cases, surgical interventions can create new nasal passageways for stent placement or even bypass the entire canalicular system.

### 3.3. Indications for Nasolacrimal Intubation

The indications for nasolacrimal intubation include nasolacrimal duct obstructions (NLDO) or lacerations within nasolacrimal pathway [123]. Patients presenting with nasolacrimal obstructions typically exhibit epiphora due to inadequate drainage. Such obstructions can occur at various points within the system, including the punctum, proximal canaliculus, common canaliculus, lacrimal sac, and nasolacrimal duct. The etiology of these obstructions is diverse, ranging from trauma, congenital and infection to malignancy and iatrogenic causes, among others [121].

One frequent surgical application of stents is post-dacryocystorhinostomy (DCR), aiming to preserve osteotomy patency [124,125]. 

### 3.4. Ideal Properties for Tear Duct Stents and Tubes

The ideal characteristics of nasolacrimal stent materials include biocompatibility, resistance to biofilm formation, and elasticity for self-expansion.

1. Biocompatibility: Nasolacrimal stent materials should be biocompatible to minimize the risk of adverse reactions and ensure proper integration with the surrounding tissues. Biocompatibility is essential for maintaining the health of the lacrimal duct and preventing complications such as inflammation and tissue damage [126]. This, in turn, reduces the likelihood of complications like granuloma formation, which is a known issue in nasolacrimal intubation.
2. Resistance to biofilm formation: Biofilm formation on the surface of nasolacrimal stents can lead to infections and other complications. Therefore, stent materials should be resistant to biofilm formation to minimize the risk of infection and ensure the long-term success of the stent [127]. Recent studies have identified a connection between infections in the nasolacrimal drainage system and biofilm formation, particularly from organisms such as nontuberculous mycobacteria [128]. Notably, advancements in stent material have been made to mitigate this risk. For instance, Shape Memory Polymer (SMP) stents have been demonstrated to exhibit superior resistance to biofilm formation when compared to traditional silicone stents [127].
3. Elasticity for self-expansion: Nasolacrimal stents should have the ability to self-expand to fit the shape and size of the lacrimal duct. Elasticity is crucial for ensuring proper tear drainage and minimizing the risk of stent migration or dislodgement. SMP stents, for instance, have demonstrated better tear drainage capacity due to their elasticity and self-expansion properties [127].

An overview of the key features, advantages and disadvantages of emerging biomaterials used in lacrimal system repair is further described in the below sections and summarized in Table 2. 

### 3.5. Biopolymers for Tear Duct Stents and Tubes

Silicone has traditionally been used for nasolacrimal duct obstruction and stenosis. However, success rates for the procedure, particularly for nasolacrimal duct stenosis, have varied widely [129,130,131]. The therapeutic landscape for nasolacrimal blockages has since expanded through the utilization of biopolymers. In the mid-1990s, the introduction of polyurethane stent insertion presented a promising option, although long-term investigations reveal a decline in its success rate over time. For instance, a 2002 study by Yazici and colleagues demonstrated that polyurethane stents achieved successful implantation in 96% of 52 eyes belonging to 49 patients afflicted with severe epiphora due to nasolacrimal duct blockage. Over a 23-month average follow-up, clinical success was realized in 69% of cases, with responsive management of lacrimal symptoms through topical treatment and irrigation. This study also brought to light the need for surgical interventions in cases of stent treatment failure, unveiling complex anatomical changes and chronic inflammation within the lacrimal sac [34]. Later, in 2005, Bertelmann and Rieck reviewed 92 patients who had undergone stent implantation, revealing that after a 5-year span, the long-term success rate was only 18.5%. Potential factors contributing to these poor long-term outcomes included stent surface structure, potential disruption of the lacrimal mucosa properties, stent design, induction of chronic inflammatory responses and the duration of stent placement [132]. Thus, both authors confirmed the unfavorable long-term viability of polyurethane. 

Baruah and collaborators studied polypropylene (Prolene; Ethicon) as a more affordable and accessible stent alternative to the standard silicon stents in DCR. Their study involved 51 cases, and their results indicated that polypropylene stenting is a promising and affordable alternative to silicone stent intubations, particularly in resource-constrained settings [133]. A recent comparative study by Ghallab and colleagues examined the effectiveness and safety of silicone to polypropylene stents in 40 patients for primarily nasolacrimal duct obstructions with or without the use of endoscopic DCR. They found that overall, silicone stents exhibited a higher success rate (95%) compared to polypropylene stents (80%), and the use of DCR during the procedure improved efficacy, although it was associated with increased complications, particularly with polypropylene stents [134].

PLLA, PCL, and polyethylene glycol (PEG) are FDA-approved biodegradable materials. However, stents made from them are typically unsuitable for lacrimal duct support because of degradation, strength, and remodeling issues. Zhan and colleagues developed and tested novel PLLA-PCL-PEG complexes for potential use in rabbit lacrimal ducts. They developed stents from these complexes in different ratios for in vitro evaluation of mechanical strength and biodegradation, then tested the best-performing ones on 32 rabbits, with half receiving the selected stents and the other half receiving silica gel stents as controls. The stent composed of PLLA:PCL6:4+5% PEG demonstrated superior biodegradability, less irritation, and quicker tissue recovery compared to the silica gel control in treating lacrimal duct obstruction disease; however, subsequent studies have yet to confirm these results [135]. 

### 3.6. Biopolymers for Lacrimal Gland Tissue Engineering

Biopolymers hold promise for creating effective healing environments that prevent inflammation and support structured lacrimal gland tissue regeneration to address dry eye disease arising from lacrimal gland insufficiency. In an earlier 2006 study, Long and colleagues tested the application of polyethersulfone (PES) dead-end tubes and membranes as a scaffold for artificial exocrine lacrimal glands, owing to their beneficial oxidative, thermal and hydrolytic stability and good mechanical and film-forming properties. Their results showed that the PES tubes permitted the passage of essential nutrients, such as ascorbic acid, L-tryptophan, and glucose to pass through, but prevented immune cell passage from entering and diminishing cell growth. The tubes also supported the attachment and growth of rat lacrimal acinar cells; however, this growth was limited [136]. Selvan and colleagues investigated the growth and functionality of purified rabbit lacrimal gland acinar cells on several matrix protein-coated polymers including silicon, collagen I, copolymers of PLGA (85:15 and 50:50), poly-L-lactic acid (PLLA), and Thermanox^®^ plastic cell culture coverslips. Their study demonstrated that PLLA best supported expression of acinar cells in comparison to other agents [137]. In a follow-up study, the same researchers used a solvent-cast/particulate leaching technique to fabricate microporous PLLA membranes from PLLA/polyethylene glycol blends. Their findings revealed the permeability of glucose, L-tryptophan and dextran (a high-molecular-weight glucose polymer) with decreasing diffusion of immunoglobulin G through the material. Cells cultured on the membrane grew to subconfluent monolayers but retained the morphological and physiological features of lacrimal acinar cells in vivo [138]. Both studies verified the potential of PLLA-based membranes as scaffolds for artificial lacrimal gland development. 

Hydrogels derived from decellularized extracellular matrix have also become a valuable substrate in tissue engineering due to their ability to enhance cell function. Wiebe-Ben Zakour and her team decellularized porcine lacrimal gland tissue and hydrolyzed it with pepsin for 12, 24, or 96 h to obtain a decellularized lacrimal gland hydrogel. Proliferation of porcine lacrimal gland epithelial cells grown on the hydrogel, Matrigel and collagen-I hydrogel were then compared. Decellularized hydrogels hydrolyzed for at least 24 h demonstrated positive epithelial cell proliferation and functionality in contrast to the Matrigel and collagen-I materials, confirming its suitability for lacrimal gland tissue engineering in an in vitro model [139]. Kaya and colleagues studied the biodegradation of a hydrogel derived from porcine decellularized lacrimal glands crosslinked with genipin, a water-soluble cross-linking reagent. They found that crosslinking the hydrogel with genipin concentrations of 0.1–5 mM was able to prevent cell degradation without negatively affecting the viability of lacrimal associated cells and the secretary capacity of epithelial cells [140]. Interestingly, using sphere-forming culture techniques and rabbit lacrimal gland progenitor cells, Lin and colleagues were able to form duct- and acinar-like lacrimal gland structures in a 3D culture system with secretory capability [141]. Similarly, Spaniol and collaborators generated a 3D cellularized lacrimal gland scaffold from porcine tissue. Secretory lacrimal cells were cultured from small biopsies and compared with enzymatic isolation, both showing epithelial characteristics, sustained functionality, and progenitor cell trains. Reseeding these cells into the decellularized lacrimal gland scaffold displayed lacrimal gland-like morphology and secretory activity, suggesting the feasibility of engineering functional 3D lacrimal gland constructs for potential therapeutic applications [142].

In another approach, Dai and collaborators created degradable in situ hydrogels tailored to tear ducts by incorporating indocyanine green fluorescent tracer nanoparticles (FTN) in a network of methacrylate-modified silk fibroin (SFMA). Silk fibroin was applied due to its hydrophobic crystalline structure, giving it strength and versatility. The SMFA/FTN hydrogels demonstrated improved lacrimal fluid retention and no inflammatory response a rabbit dry eye model with no inflammatory response, making potential advancements in this field [143].

Chitosan, sourced from crustacean exoskeletons, is valued for biocompatibility, biodegradability, antibacterial properties, and wound-healing capabilities, positioning it as another optimal choice for biomedical applications like lacrimal gland tissue repair. Studies reveal that chitosan encourages gland development and hepatocyte growth factor (HGF) binding, a protein essential in promoting cell growth and tissue development. For instance, Hsia and Yang’s conducted experiments involving embryonic lacrimal gland explants, wherein chitosan exhibited a dose-dependent stimulation of branching morphogenesis, with optimal results observed at a concentration of 0.3 mg/mL. Moreover, chitosan amplified the in vivo binding affinity of HGF-related molecules. However, the introduction of inhibitors, such as PD98059 (targeting the MAPK pathway) and LY294002 (targeting the Akt/PKB pathway), annulled chitosan’s branching-promoting effects [144,145].

Recently, 3D bioprinting has also emerged as a promising method in tissue engineering, allowing for the biofabrication and replication of complex biological tissues. Rodboon and colleagues showed the ability of a magnetic 3D bioprinting (M3DB) system for 3D in vitro biofabrication of cellularized tissues using magnetic nanoparticles to bring cells together. Their preliminary research using M3DB to create lacrimal gland organoids from murine and porcine primary cells has shown that this approach yields strong organoids suitable for future high-throughput analysis and drug discovery. They concluded that the lacrimal gland organoids have the potential to serve as a functional model of tear production, offer a platform for drug screening, and possibly for clinical applications in treating dry eye disease [146].

**Table 2:** Emerging Biomaterials in Lacrimal System Repair.

Material Type	Key Features	Advantages	Challenges	References
Polyurethane	-Stents to manage blocked lacrimal duct	-Up to 96% success rate observed in clinical studies -Comparable results of external dacryocystorhinostomy (DCR)	-Unfavourable long-term viability -Potential factors contributing to poor long-term outcomes include stent surface structure, disruption of lacrimal mucosa properties, stent design, induction of chronic inflammatory responses, and the duration of stent placement -Surgical interventions may be needed in cases of stent treatment failure	[34,132,147,148]
Polypropylene	-Cost-effective alternative to silicone stents in DCR	-Cheaper and more readily available than silicone -Potential option in resource-constrained settings	-Lower success rate compared to silicone	[133,134]
PLLA, PCL and polyethylene glycol (PEG) complexes	-FDA-approved biodegradable material for lacrimal duct repair	-Some stents have demonstrated superior biodegradability, less irritation and quicker tissue recovery compared to silical gel stents -Initial studies show promising results	-Lack of subsequent research confirming effectiveness -Stents made from these materials have typically been considered unsuitable for lacrimal duct support due to degradation, strength, and remodeling issues	[135]
Polyethersulfone (PES)	-PES dead-end tubes and membranes applied as scaffold for artificial exocrine lacrimal glands	-Beneficial oxidative, thermal, and hydrolytic stability -Good mechanical and film-forming properties -Allow passage of nutrients like ascorbic acid, L-tryptophan, and glucose while preventing immune cell entry and diminishing cell growth -Tubes supported the attachment and growth of lacrimal acinar cells in rat models	-Limited application due to challenges in achieving robust cell growth and development -Lack of human studies	[136]
PLLA-based materials	-Purified rabbit lacrimal gland acinar cells on various matrix protein-coated polymers has been studied for lacrimal tissue engineering -Included silicon, collagen I, poly-D,L-lactide-co-glycolide (PLGA; 85:15 and 50:50), poly-L-lactic acid (PLLA), and Thermanox^®^ plastic cell culture coverslips	-PLLA found to best support expression of acinar cells compared to other agents making it a promising candidate for lacrimal gland scaffolds	-While PLLA-based membranes show permeability to glucose, L-tryptophan, and dextran, they demonstrated decreased diffusion of immunoglobulin G	[137,138]
Hydrogels from decellularized and cellular tissues	-Formulated hydrogel gel for lacrimal gland tissue engineering	-Positive epithelial cell proliferation and functionality in contrast to other materials -Crosslinking hydrogels with genipin can prevent degradation without negatively affecting the viability of lacrimal-associated cells and the secretary capacity of epithelial cells -3D culture systems using hydrogels and lacrimal gland progenitor cells have shown the formation of duct- and acinar-like lacrimal gland structures with secretory capability -Cellularized lacrimal gland scaffolds derived from porcine tissue have been generated and displayed lacrimal gland-like morphology and secretory activity when reseeded with lacrimal cells	-Lack of in vivo studies -Additional research required to assess safety and efficacy	[139,140,141,142]
SFMA/FTN hydrogen plug	-In situ hydrogel tailored for tear ducts -Incorporate indocyanine green fluorescent tracer nanoparticles (FTN) within a network of methacrylate-modified silk fibroin (SFMA)	-Silk fibroin chosen for its hydrophobic crystalline structure, providing strength and versatility -Improved lacrimal fluid retention with no inflammatory response in rabbit dry eye model	-Further research and testing needed to validate the safety and efficacy of these hydrogels	[143]
Chitosan	-Sourced from crustacean exoskeletons	-Valued for its biocompatibility, biodegradability, antibacterial properties, and wound-healing capabilities -In experiments involving embryonic lacrimal gland explants, chitosan exhibited a dose-dependent stimulation of branching morphogenesis, with optimal results observed at a 0.3 mg/mL concentration -Amplified the in vivo binding affinity of HGF-related molecules, contributing to tissue development	-Use of inhibitors targeting specific pathways, such as PD98059 (MAPK pathway) and LY294002 (Akt/PKB pathway), can annul chitosan’s branching-promoting effects -Limited research to date	[144,145]
3D bioprinting techniques	-Replication of biological tissues -Magnetic 3D bioprinting (M3DB) utilizes magnetic nanoparticles to bring cells together for 3D in vitro biofabrication of cellularized tissues	-M3DB has shown potential in creating lacrimal gland organoids from murine and porcine primary cells, resulting in strong organoids suitable for high-throughput analysis and drug discovery -Lacrimal gland organoids can serve as a functional model for tear production, a platform for drug screening, and may have clinical applications in treating dry eye disease	-While the preliminary research is promising, further studies are needed to validate the safety, efficacy, and clinical potential of lacrimal gland organoids created through M3DB	[146]

### 3.7. Challenges, Barriers, Gaps in Knowledge, and Future Directions

The use of biopolymers in lacrimal duct stents and lacrimal gland tissue engineering presents both challenges and opportunities. While traditional silicone stents have variable success rates in treating nasolacrimal blockages, newer biopolymers like polypropylene show promise, particularly in resource-constrained settings. However, long-term viability remains a challenge, with factors like surface structure, mucosa properties disruption, and chronic inflammation affecting outcomes. On the other hand, biodegradable materials like PLLA, PCL, and PEG may offer potential solutions but require further confirmation.

Bioengineered lacrimal glands have demonstrated the ability to functionally restore tear secretion and protect the ocular surface, offering promise for dry eye disease and xerostomia therapies. Specifically, biopolymers like polyethersulfone, PLLA, and decellularized hydrogels have shown potential in supporting cell growth and functionality. Notably, hydrogels derived from decellularized ECM and chitosan have demonstrated possible lacrimal gland regeneration due to their biocompatibility and modulatory effects on cellular functions. However, their precise control and full potential remain not fully understood. Currently available hydrogel products for tissue engineering are limited, but as medicine advances, in-situ adaptable hydrogels may become a breakthrough biomaterial with multiple functions tailored to individual patients. Research is progressing towards selecting more effective polymers and bioactive substances based on chemical structure-based properties and introducing stimulus-responsive and mechanical properties through molecular design. Comprehensive evaluation and the development of disease-like animal models are expected to expedite clinical applications of hydrogels in tissue regeneration [149]. The integration of 3D bioprinting technologies also holds exciting prospects for creating functional lacrimal gland organoids for drug screening and potential clinical applications [146]. Further, stem cells, including pluripotent stem cells, hold potential for organ regeneration, but research is needed to explore cell sources, disease models, culture methods, and transplantation techniques for practical clinical applications [150]. 

Overall, these studies underscore the complexity of selecting the right biopolymer for lacrimal duct and gland applications, emphasizing the need for a balance between short-term effectiveness and long-term success in these therapies.

## 4. Conclusions

In conclusion, this review encapsulates the progressive intersection of biopolymer science and oculoplastic clinical practice, shedding light on the sophisticated applications of biopolymers in reconstructing the posterior lamella of the eyelid and the lacrimal system. It accentuates the multifaceted role of these materials in addressing the anatomical and functional intricacies of periocular structures. The literature review from 2015 to 2023 presented herein not only bridges the knowledge gap but also serves as a testament to the dynamic nature of surgical biomaterials research—a field that is as diverse in its challenges as it is in its potential to revolutionize patient care.

The novelty of our review is underscored by its amalgamation of clinical acumen with material science. It transcends the traditional scope of literature by emphasizing the translational pathway of these biomaterials, advocating for a multidisciplinary collaboration that is quintessential for the actualization of benchtop discoveries in clinical settings. The insights offered by clinicians enrich the narrative, ensuring that the dialogue is not only theoretically robust but also pragmatically relevant, with a clear vision toward enhancing patient outcomes.

Ultimately, this review underscores the necessity of an integrative approach, where the convergence of expertise from material scientists, engineers, and clinicians catalyzes the development of solutions that are not only innovative but also compassionate. It is this synergy that will propel the field of ophthalmology towards a future where the quality of life for patients is not just preserved but significantly improved. The potential for these advancements to redefine the landscape of ocular surgery is immense, promising a horizon where the restoration of sight is matched by the resilience of science and the humanity of care.

## Data Availability

Not applicable.
